# Volatile Fatty Acids (VFAs) Generated by Anaerobic Digestion Serve as Feedstock for Freshwater and Marine Oleaginous Microorganisms to Produce Biodiesel and Added-Value Compounds

**DOI:** 10.3389/fmicb.2021.614612

**Published:** 2021-01-28

**Authors:** Alok Patel, Amir Mahboubi, Ilona Sárvári Horváth, Mohammad J. Taherzadeh, Ulrika Rova, Paul Christakopoulos, Leonidas Matsakas

**Affiliations:** ^1^Biochemical Process Engineering, Division of Chemical Engineering, Department of Civil, Environmental, and Natural Resources Engineering, Luleå University of Technology, Luleå, Sweden; ^2^Swedish Centre for Resource Recovery, University of Borås, Borås, Sweden

**Keywords:** omega-3 fatty acids, biofuels, microalgae, oleaginous microorganisms, volatile fatty acids

## Abstract

Given an increasing focus on environmental sustainability, microbial oils have been suggested as an alternative to petroleum-based products. However, microbial oil production relies on the use of costly sugar-based feedstocks. Substrate limitation, elevated costs, and risk of contamination have sparked the search for alternatives to sugar-based platforms. Volatile fatty acids are generated during anaerobic digestion of organic waste and are considered a promising substrate for microbial oil production. In the present study, two freshwater and one marine microalga along with two thraustochytrids were evaluated for their potential to produce lipids when cultivated on volatile fatty acids generated from food waste via anaerobic digestion using a membrane bioreactor. Freshwater microalgae *Auxenochlorella protothecoides* and *Chlorella sorokiniana* synthesized lipids rich in palmitic acid (C16:0), stearic acid (C18:0), oleic acid (C18:1), and linoleic acid (C18:2). This composition corresponds to that of soybean and jatropha oils, which are used as biodiesel feedstock. Production of added-value polyunsaturated fatty acids (PUFA) mainly omega-3 fatty acids was examined in three different marine strains: *Aurantiochytrium* sp. T66, *Schizochytrium limacinum* SR21, and *Crypthecodinium cohnii*. Only *Aurantiochytrium* sp. T66 seemed promising, generating 43.19% docosahexaenoic acid (DHA) and 13.56% docosapentaenoic acid (DPA) in total lipids. In summary, we show that *A. protothecoides*, *C. sorokiniana*, and *Aurantiochytrium* sp. T66 can be used for microbial oil production from food waste material.

## Introduction

A green and sustainable bio-based economy has become a key element of long-term growth and well being. Both developed and developing countries have committed to a transition from petroleum-based industries to the manufacturing of more sustainable and renewable materials (Cho and Park, 2018). This puts a strong incentive on commercially feasible processing of low-cost renewable substrates for industrial production of bio-based chemicals. The oleochemical industry relies mostly on vegetable oils and animal fats as raw materials; however, concerns about food security and sustainability have called for alternatives. Oil derived from microbial cultivation benefits from high biomass and lipid productivities, independence from external climatic conditions, and shorter production cycle than plants (Patel et al., 2020a). Moreover, given a similar fatty acid profile and properties as those of plant oils, it could offer a sustainable alternative to the latter.

Microalgae, bacteria, fungi, and yeast are considered oleaginous microorganisms, in which lipids can constitute more than 20% w/w of their cellular biomass (Papanikolaou, 2012). In some species, lipids can make up to 70% w/w of cell dry weight, particularly with cultivation at a high C/N ratio (Papanikolaou and Aggelis, 2011). Most lipids accumulated by oleaginous microorganisms possess 4–28-long unbranched carbon chains (Dewick, 2009). Depending on the number of double bonds, they can be either monounsaturated or polyunsaturated (Ratledge, 2004). The high proportion of polyunsaturated fatty acids (PUFAs) in lipids derived from oleaginous microorganisms makes them unsuitable as biodiesel substrates because the presence of more than two double bonds promotes unwanted oxidation (Knothe, 2012; Patel et al., 2019a, 2018). Instead, these PUFAs could be used as an energy-rich dietary source of docosahexaenoic acid (DHA) and eicosapentaenoic acid (EPA) by humans, as we lack the desaturases and elongases required to synthesize them (de Jong et al., 2014; Lands, 2014; Zárate et al., 2017). Both DHA and EPA have various importance in metabolic and immune activities and crucial role in health benefits related to neuro and cardiovascular diseases (Kris-Etherton et al., 2010; Flock et al., 2013). Fish oils are the most readily available source of these dietary fatty acids, but diminishing aquatic resources and increasing demand for omega fatty acids mean that fish cannot remain a long-term feedstock (Hamilton et al., 2020; Katerina et al., 2020). Hence, oleaginous microorganisms could provide a sustainable source of EPA and DHA. The global market for microalgae-based DHA was valued at $350 million in 2012 and was revised upward to $4,212 million in 2017, indicating a clear spike in demand for superior-quality microalgal DHA (Subhadra and Edwards, 2011; Vigani et al., 2015; Bannenberg et al., 2017). In contrast with phototrophic conditions, heterotrophic conditions will increase the concentration of biomass by as much as 25-fold (Morales-Sánchez et al., 2017). Currently, these types of cultivation are economically feasible only for high-value products such as PUFA, pigments, antioxidants, polysaccharides, food and aquaculture feed from carbon sources, such as glucose, acetate or glycerol (Morales-Sánchez et al., 2017). Instead of using pure substrates in heterotrophic cultivation, the substrates obtained from low-cost non-edible lignocellulosic biomass, agricultural residues, and other waste substrates bring down the overall production cost (Patel et al., 2016). Enzymatic hydrolysis of these carbohydrate-rich waste substrates to generate feedstock for oleaginous microorganisms and for the generation of omega-3 fatty acids has been a promising way of valorizing such resources.

A more cost-effective alternative to enzymatic hydrolysis is anaerobic digestion (AD), which is traditionally used for biogas production. However, AD has recently been developed to convert a wide range of organic waste materials with different macromolecular composition (carbohydrates, lipids, and proteins) to volatile fatty acids (VFAs) (Lukitawesa et al., 2020). Hydrolysis, acidogenesis, acetogenesis, and methanogenesis are the four different degradation stages of anaerobic digestion, during which organic material is converted to biogas (Anukam et al., 2019). After the hydrolytic and acidogenic stages, the generation of VFAs as intermediates proceeds in an entirely sustainable way. VFAs are short-chain (C2–C6) organic acids that serve as a carboxylate platform for building blocks to be used in the chemical industry (Llamas et al., 2020). Besides being a feedstock for biofuels (Choi et al., 2011) and bioplastic production (Mengmeng et al., 2009), VFAs can be applied for conversion into alcohols, aldehydes (Spirito et al., 2014), bioelectricity (Béligon et al., 2015). In recent years, sustainability demands have favored the biological route based on the use of pure sugar substrates for commercial processing of VFAs (Kondo and Kondo, 1996; Huang et al., 2002; Akaraonye et al., 2010). However, this poses some ethical issues regarding the usage of food for chemicals. To overcome this dilemma, food substrates should be replaced with other organic-rich waste materials, such as sludge derived from food waste, municipal solid waste, and industrial water.

The most common VFAs are acetic (C2), propionic (C3), isobutyric, butyric (C4), isovaleric, valeric (C5), and caproic (C6) acids (Wainaina et al., 2019). The different ratio of VFAs produced depends on operational conditions, substrate composition, and microbial population in the anaerobic digestion system (Lukitawesa et al., 2020). Mixtures of VFAs are less valuable, unless they are turned into added-value chemicals or purified as a single component. The separation and purification of VFAs is difficult because they form an azeotropic mixture with H_2_O, which is not compatible with subsequent chemical platforms (Woo and Kim, 2019). In comparison, oleaginous microorganisms can directly convert some organic acids into acetyl coenzyme A (CoA) via fatty acid degradation and CoA synthetase. CoA is a central intermediate in lipid synthesis, including that of PUFAs in oleaginous cells (De Swaaf et al., 2003b; Sijtsma and De Swaaf, 2004).

Study on lipogenesis by oleaginous microalgae and yeast cultivated on single VFA as well as on the mixture of VFAs are extensively studied for biofuel production (Xia and Murphy, 2016; Llamas et al., 2020). PUFA production by marine microalga *Crypthecodinium Cohnii* cultivated on mixture of VFAs is also an explored topic (Chalima et al., 2017), but to the best of our knowledge this is the first attempt to cultivate an oleaginous marine thraustochytrids using a mixture of VFAs as carbon source. Moreover, in this study, five oleaginous microorganisms were selected to cultivate on VFAs produced through anaerobic digestion of food mixture.

There are many advantages to the anaerobic digestion of food waste, including, mitigation of climate change, economic benefits, and diversion opportunities (Xu et al., 2018). In a LCA analysis, it was mentioned that by replacing approximately 9,900 t of corn silage with 6,600 t of food waste, almost 42% reduction in CO_2_ emission of the electricity produced from the biogas plant could be achieved (Bartocci et al., 2020). Slorach et al. (2020) suggested that Anaerobic digestion is environmentally the most sustainable option with lowest overall impact on the food-energy-water-health nexus (Slorach et al., 2020). Food waste after landfilling produces methane as a potent greenhouse gas. The transfer of food waste from landfills to wastewater treatment plants, enable to capture the generated methane for renewable energy purposes and at the same time reduces the greenhouse gas emissions due to the energy offsets provided by using an on-site, renewable source of energy (Awasthi et al., 2020). Wastewater treatment facilities should anticipate seeing efficiency gains from the combination of anaerobic digesters with food waste that included lower energy cost due to on site power generation. The majority of municipalities invest in means of diverting materials from landfills. This is generally attributed to diminished landfill space and/or targets for recycling. Wastewater treatment plants provide the ability to divert vast volumes of food waste, one of the biggest waste sources currently going to landfills worldwide (Awasthi et al., 2020).

## Materials and Methods

### VFA Production via Anaerobic Digestion in a Membrane Bioreactor

The membrane bioreactor used for the anaerobic digestion of food waste consisted of a continuously stirred tank reactor with a 2nd generation microfiltration integrated permeate channel membrane panel (PES, 0.3 μm pore size; VITO NV), with a filtration area of 68.6 cm^2^ submerged in a continuous stirred tank reactor (bbi biotech GmbH, Germany) and with 2 L working volume. The use of this membrane bioreactor for semi-continuous fermentation, as well as VFA production and recovery was described previously by Parchami et al. (2020). The reactor was equipped with a flow meter, pressure sensor, relay (for inversion of flow direction during backwashing), and peristaltic permeate pump. The latter served to collect the effluent VFA solution and track changes in membrane filtration performance through permeate flux and transmembrane pressure recordings. Membrane cleaning and anti-fouling measures were implemented by intermittent nitrogen sparging and backwashing. During the course of the experiment, 200 mL of particle-free reactor medium was filtered out per day and replaced by food waste.

The substrate used to generate VFAs through anaerobic digestion was a model mixture of food waste from the European Union (Ariunbaatar et al., 2016) composed of fruits and vegetables, pasta and rice, bread and bakery products, meat and fish, and dairy. The initial feed mixture contained 16.11 ± 0.98% total solids, 15.41 ± 0.94% volatile solids, and 60.00 ± 5.66 g/L soluble chemical oxygen demand. The membrane bioreactor was inoculated with an inoculum derived from an anaerobic sludge blanket reactor used for wastewater treatment (Hammarby Sjöstad, Stockholm, Sweden), containing 9.55 ± 0.35% total and 6.48 ± 0.25% volatile solids.

### Microorganisms and Cultivation Conditions

Five different oleaginous microorganisms were utilized for the cultivation experiments: the two thraustochytrids *Aurantiochytrium* sp. T66 ATCC-PRA-276 (PRA) and *Schizochytrium limacinum* SR21 ATCC-MYA-1381 (SR21), the marine microalga *C. cohnii* PGM-1 ATCC-30772 (Cohnii), and the two freshwater microalgae *Chlorella sorokiniana* SAG 211-8k (CS) and *Auxenochlorella protothecoides* SAG 211-13 (AP). The first three were obtained from the American Type Culture Collection (ATCC) and the latter two from the culture collection of algae (SAG) at Göttingen University, Germany. PRA, SR21, and Cohnii were initially grown in ATCC^®^ 790 By + medium containing yeast extract (1 g/L), peptone (1 g/L), glucose (5 g/L), and seawater (1,000 mL). The freshwater microalgae AP and CS were cultivated on bold modified basal freshwater- nutrient solution (BBM; Sigma-Aldrich B5282-500 mL), which included glucose (20 g/L) and yeast extract (C/N 20). The pH of both media was adjusted to 6.8 and cultivations were carried out in 250 mL Erlenmeyer flasks, with 100 mL working solutions at 25°C in an incubator shaker at 180 rpm.

### Batch Cultivation of Microorganisms on VFAs Produced From Anaerobically Digested Food Waste

Media for cultivation of freshwater and marine microorganisms were prepared separately and were mixed with a VFAs solution. The VFAs solution consisted of acetic acid (C2; 2.75 g/L), propionic acid (C3; 1.43 g/L), butyric acid (C4; 1.41 g/L), valeric acid (C5; 0.25 g/L), carponic acid (C6; 4.78) g/L) which contained 10.92 g/L of total carbon source, together with a small amount of ammonium (0.3 g/L). Marine microorganisms (PRA, SR21, and Cohnii) were first cultivated in a VFAs solution with C/N of 10 to assess the effect on biomass and lipid accumulation. Then, they were shifted to C/N of 20. The C/N ratio was calculated based on total carbon and nitrogen present in the VFAs solution and was adjusted with an appropriate amount of yeast extract followed by addition of sea salt (15 g/L). The volume was adjusted to 90% of the final volume and the pH was set to 6.8 with 3 mol/L NaOH and 3 mol/L HCl. Cultivation experiments were carried out in 250 mL Erlenmeyer flasks with 100 mL of working solution. Seed culture (10%, v/v) was used to inoculate the medium and the flasks were incubated in orbital shaker with 180 rpm at 25° C until stationary phase was achieved. Freshwater microalgae (AP and CS) were cultivated on a VFAs solution with BBM at C/N of 20 and C/N of 60 adjusted with yeast extract. The pH of both media was set to 6.8 with 1 M NaOH and 1 M HCl, while 100 mM Tris(hydroxymethyl)aminomethane was added to maintain medium pH during cultivation. Experiments were carried out in 250 mL Erlenmeyer flasks with 100 mL working solution. The medium was inoculated with 10% of a seed culture and the flasks were incubated at 25°C in an orbital shaker at 180 rpm until stationary phase was achieved.

### Cell Growth and Estimation of VFAs Consumed During Fermentation

Samples (2 mL) were collected after 24 h of cultivation and optical density was measured at 680 nm with a UV-Vis spectrophotometer (Molecular Devices Spectra Max M2). To determine VFAs consumption, high-performance liquid chromatography (HPLC) was carried out on the supernatant from the collected samples after filtration through a 0.2 μm syringe filter (Sartorius^TM^ Minisart^TM^ RC) into HPLC vials. The HPLC apparatus (Perkin Elmer Series 200) was equipped with a Bio-Rad Aminex HPX-87H column (#1250140) and programmed for 30 min with 5 mM H_2_SO_4_ as mobile solvent. The column temperature was set to 65°C and a refractive index detector was used to detect the peaks of C2, C3, and C4 VFAs. The peaks of C5 and C6 VFAs were determined on the same HPLC column and instrument but using a different program from the Bio-Rad Bulletin 1928 Rev B. The HPLC was programmed for 50 min with 5 mM H_2_SO_4_ and acetonitrile (90:10;% v/v) as mobile phase, and peaks were detected at 210 nm. Identification and quantification of VFAs were performed with calibration curves prepared with synthetic VFA standards.

### Cell Dry Weight and Total Lipid Estimation

Once cultures entered stationary phase, the cells were harvested by centrifugation (Eppendorf 5804 R with a F-34-6-38 rotor) at 8,000 rpm (7,881 × *g*). The pellets were kept in pre-weighed pans and dried in a hot-air oven at 40°C until weight was constant. The cell dry weight in g/L was determined gravimetrically. The supernatant was used for the determination of residual carbon sources in the medium by HPLC. Dried biomass was used to extract the lipids. The biomass was crushed into a fine powder with a mortar and pestle, the powder was blended with chloroform: methanol (2:1), and incubated for 2 h with shaking. Subsequently, deionized water was added to the slurry. The volume of added water equaled half the volume of the slurry. The tube was mixed thoroughly and centrifuged at 8,000 rpm (7,881 × *g*) for 10 min. The bottom clear phase was aspirated in a pre-weighed watch glass and placed into a hot-air oven at 50°C to evaporate the solvent. The watch glass with the dry lipids was weighed again and stored in a freezer at −20°C for further analysis.

### Assessment of Lipid Accumulation During Cultivation Through Fluorescence Microscopy

Morphological analysis and estimation of lipid synthesis during cultivation of AP, CS, PRA, SR21, and Cohnii on VFAs were monitored through fluorescence microscopy. 1mL of samples were drawn out from growing culture at their early stationary phase, followed by three time washing to remove medium components, and suspended in 100 μL of 0.9% saline solution. BODIPY_493__/__503_ (4,4-difluoro-1,3,5,7,8-pentamethyl-4-bora-3a,4a-diaza-s-indacene) stock solution was prepared with the concentration of 0.1 mg/mL of DMSO. BODIPY solution (2 μL) was added to the 100 μL samples and were incubated for 5 min in the dark. The imaging was performed on a digital inverted fluorescence microscope equipped with a GFP light cube (EVOS-FL, Thermo Fisher Scientific).

### Assessment of Fatty Acid Profile by Gas Chromatography-Mass Spectrometry (GC-MS)

The obtained lipids were transesterified with an acid-based catalyst as described previously (Van Wychen et al., 2013). Initially, lipids (50–100 mg) were dissolved in chloroform:methanol (2:1, v/v) inside an ace pressure tube, (Sigma-Aldrich), after which 3 mL of 0.6 M HCl:methanol was added to the mixture. The tubes were placed in a preheated water bath at 85°C for 1 h. Next, *n*-hexane (3 mL) was added to the mixture after cooling at 25°C. The mixture was centrifuged at 8,000 rpm (7,881 × *g*) for 10 min to separate the various layers. The upper *n*-hexane layer containing fatty acid methyl esters (FAMEs) was aspirated and transferred to new GC vials for analysis on a GC-MS system (Clarus 690 coupled to Clarus SQ8; PerkinElmer) equipped with a capillary column (Elite 5MS; 30 m, 0.25 mm ID, 0.25 μm df, # N9316282; PerkinElmer). GC-MS analysis was performed as described previously by Patel et al. (2020c). The oven was programmed to 50°C for 0.50 min, temperature was ramped to 194°C at 30°C/min for 3.50 min, and then to 240°C at 5°C/min, where it was held for 10 min. The injection port temperature was adjusted at 250°C and 1 μL sample was injected with He as carrier gas in split mode (10:1). Solvent delay time was 3 min. The MS transfer line temperature was adjusted to 250°C with 170°C source temperature. Mass spectra (mass range 50–400 m/z) were recorded at 3 scans/s with electron ionization at 70 eV.

## Results and Discussion

### Batch Cultivation of Freshwater Microalgae at C/N Ratios of 20 and 60

Acetate and butyrate can be used as sole carbon sources for the heterotrophic and mixotrophic cultivation of oleaginous microalgae (Patel et al., 2021). Whereas several studies have suggested that acetate is the preferred source (Turon et al., 2015b), if both are provided in the cultivation medium, butyrate inhibits the uptake of acetate (Turon et al., 2015a). Therefore, to evaluate microalgal production under heterotrophic conditions, the capacity to grow not only on single VFAs but also on a mixture of VFAs must be investigated (Turon et al., 2016). Here, the effluent from anaerobically digested food waste in a membrane bioreactor served as the VFAs mixture and its composition was analyzed by HPLC (Table 1).

**TABLE 1:** VFAs produced after anaerobically digested food waste in a membrane bioreactor.

**Type of VFAs**	**Concertation (g/L)**
Acetic acid (C2)	2.75
Propionic acid (C3)	1.43
Butyric acid (C4)	1.41
Valeric acid (C5)	0.25
Caproic acid (C6)	4.78
Total VFAs	10.62

**FIGURE 1:**
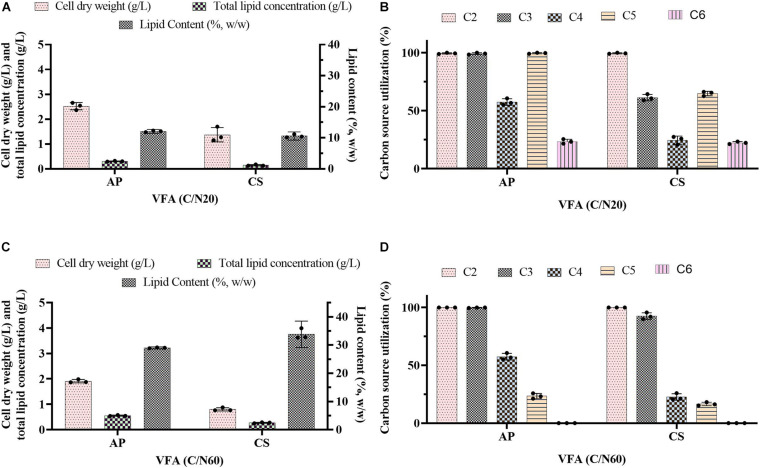
Batch cultivation of *A. protothecoides* SAG 211-13 (AP) and *C. sorokiniana* SAG 211-8k (CS) on a VFAs mixture at **(A,B)** C/N 20 and **(C,D)** C/N 60. **(A,C)** Estimation of cell dry weight (g/L), total lipid concentration (g/L), and lipid content (%, w/w). **(B,D)** Estimation of residual carbon source in the medium (g/L). Values represent the average ± standard deviation.

**FIGURE 2:**
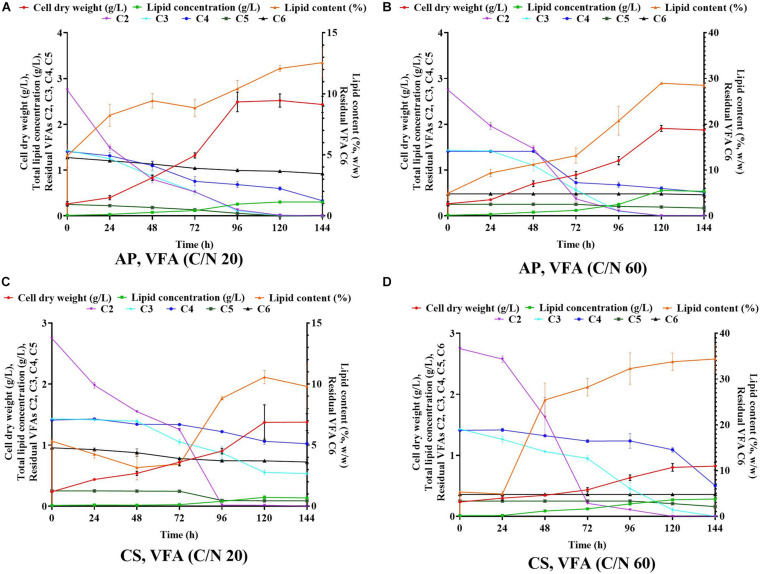
Time course experiment for cell dry weight, lipid concentration, lipid content and residual VFAs when *A. protothecoides* SAG 211-13 (AP) cultivated on VFAs at C/N 20 **(A)** and C/N 60 **(B)** and *C. sorokiniana* SAG 211-8k (CS) cultivated on VFAS at C/N 20 **(C)** and C/N 60 **(D)**.

**FIGURE 3:**
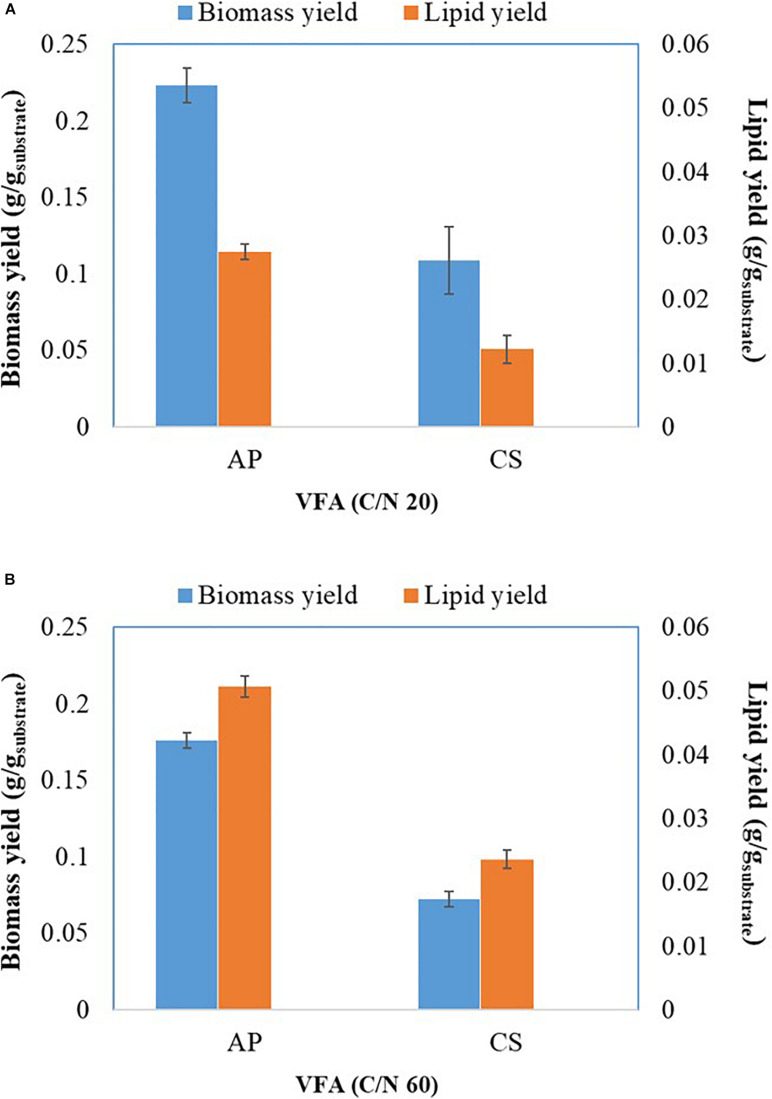
Biomass and lipid yield of *A. protothecoides* SAG 211-13 (AP) and *C.sorokiniana* SAG 211-8k (CS) cultivated on VFAs at C/N 20 **(A)** and C/N 60 **(B)**.

**FIGURE 4:**
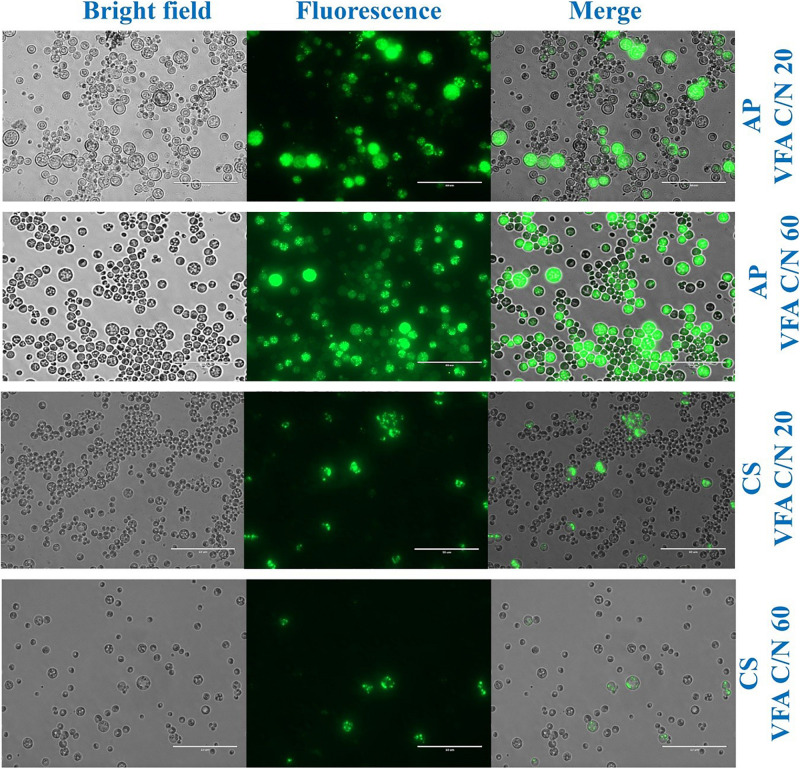
Morphologic analysis of cells and lipid droplets of *A. protothecoides* SAG 211-13 (AP) and *C. sorokiniana* SAG 211-8k (CS) cultivated on VFAs at C/N 20 and C/N 60. The cells were stained with 4,4-difluoro-1,3,5,7,8-pentamethyl-4-bora-3a,4a-diaza-s-indacene (BODIPY_493__/__503_) and observed by live fluorescence microscopy. Scale bars corresponds to 50 μm.

Previous evidence suggested that cultivation of microalgae at C/N of 20 favored biomass accumulation, whereas C/N of 60 supported lipid synthesis (Patel et al., 2018). Hence, the algal strains were cultivated on a mixture of VFAs at two different C/N ratios of 20 and 60. Cell dry weight, total lipids concentration, and lipid content obtained in these experiments are presented in Figures 1A,C, respectively, while the corresponding VFAs utilization is presented in Figures 1B,D. The time course experiment of cell dry weight, lipid concentration, lipid content and residual VFAs during the cultivation of AP and CS under C/N 20 and C/N 60 are presented in Figure 2. The AP cultivated on VFAs at C/N of 20 achieved 2.52 g/L of cell dry weight and 0.30 g/L of lipids, which corresponded to 12.08% w/w lipid content after consumption of almost all C2, C3, C5, and small amounts of C4 and C6 (Figure 2A). Highest cell dry weight and lipid concentration were achieved at 96 h of cultivation (2A). When cultivation was shifted to C/N of 60, the production of biomass decreased to 1.91 g/L, but the cells produced a higher amount of lipids (0.55 g/L), corresponding to 28.97% of lipid content (Figure 2B). Only C2 and C3 were totally utilized, whereas C5 and C6 were not (Figure 2B). The highest cell dry weight and lipid concentration were observed at 120 h of cultivation where the stationary phase was shifted from 96 to 120 h as it was at 96 h in the case of C/N 20 (Figures 2A,B). In the case of CS, cell dry weight reached 1.37 and 0.80 g/L, when cultivated on VFAs at C/N of 20 and 60, respectively, while the corresponding lipid concentration was 0.14 and 0.27 g/L. The highest cell dry and lipid concentration were reported at 120 h of cultivation in both cases of C/N 20 and C/N 60 (Figures 2C,D). The VFAs utilization pattern were totally different as reported in the case of AP. When CS was cultivated in VFA C/N 20, only 27.65% of C4 and 22.36% of C6 were utilized from the provided medium at 120 h of cultivation, while at C/N 60 it was only 22.58% of C4 and no utilization of C6 was reported (Figure 2D). Although a higher C/N ratio favored lipid accumulation in CS, the obtained cell dry weight and lipid concentration were lower compared to those reported with AP, which might be explained by lower consumption of VFAs as a carbon source (Figures 1B,D, 2). In the case of AP cultivated on VFA at C/N 20, the biomass and lipid yield were 0.22 and 0.03 g/g_substrate_, respectively, while corresponding values at C/N 60 were 0.17 and 0.05 g/g_substrate_ (Figures 3A,B). Both values were observed lower when CS cultivated on C/N 20 (Biomass yield, 0.10 g/g_substrate_ and lipid yield, 0.012 g/g_substrate_) and C/N 60 (Biomass yield, 0.07 g/g_substrate_ and lipid yield, 0.02 g/g_substrate_), respectively (Figures 3A,B). Both AP and CS cells grown on VFA at C/N 20 and C/N 60 were observed under a fluorescence microscope and the images presented in Figure 4. Both small and large cells were observed in the case of AP grown on VFAs at C/N 20, where larger cells were filled with tiny lipid droplets (Figure 4), while the cells were uniform in case of C/N 60 and all have lipid droplets inside their cellular compartment. It is already reported that CS has lower biomass and lipid accumulation than those reported with AP that is clear with the morphological analysis of CS, where only few cells showed lipid accumulation in both C/N 20 and C/N 60. Most of cells were smaller than those reported with AP (Figure 4).

Lipid accumulation in oleaginous microorganisms is affected mostly by feedstock type and concentration, temperature, pH, aeration and agitation, and uptake of nutrients (Patel et al., 2017b). A comparison of various oleaginous microorganisms cultivated on mixture of VFAs for biomass and lipid accumulation is presented in Table 2. Hu et al. (2013) suggested that a high concentration of VFAs inhibited the microalga *Chlorella* sp., which prompted cultivation in 8-fold diluted acidogenic swine effluents containing a mixture of acetate, propionate, and butyrate (Hu et al., 2013). Inhibition of microalgal growth at high concentrations of VFAs has been reported also by Liu et al. (2013a), who observed an inhibitory effect on mixotrophic growth of *Chlorella vulgaris* at more than 0.1 g/L of C4 (Liu et al., 2013a). The VFA ratio of acetic acid:propionic acid:butyric acid is another crucial factor for the growth of any microorganism and it could be altered through the VFA platform. A VFA ratio of 8:1:1 supports maximum biomass (0.65 g/L) and lipid production (0.317 g/L), both of which are lower at a VFA ratio of 4:3:3 (Fei et al., 2015). Moon et al. (2013) performed cultivation experiments of *Chlamydomonas reinhardtii* on different organic carbon sources (acetate, glucose, glycerol, and sucrose). As an alternative to acetate, they additionally tested the use of volatile fatty acids (VFAs; acetic, propionic, and butyric acids), which can be inexpensively produced through fermentation of food waste (Moon et al., 2013). Cultivation of *Chlamydomonas reinhardtii* at a 8:1:1 VFA ratio caused growth inhibition by propionic and butyric acid at elevated total VFA concentration (Moon et al., 2013). An important outcome of this study was the high amount of FAMEs generated when cells were cultivated on 5 g/L of VFAs mixture compared to 10 g/L of acetate alone under mixotrophic conditions (Moon et al., 2013). In a heterotrophic cultivation experiment, *C. protothecoides* achieved 0.50 g/L of cell dry weight when cultivated on waste activated sludge containing 3,840 mg/L chemical oxygen demand, including acetic acid (1.2 g/L), propionic acid (0.45 g/L), butyric acid (0.23 g/L), isobutyric acid (0.24 g/L), valeric acid (0.36 g/L), and isovaleric acid (0.14 g/L) (Wen et al., 2013). At a 6:1:3 ratio, acetate is the most prevalent compound (Ryu et al., 2015). Chandra et al. (2015) suggested that propionate was more complex than acetate and butyrate, as it persisted in the medium for a longer time and at a higher concentration, thus lowering the pH and inhibiting growth (Chandra et al., 2015). Acetic acid is preferred over propionic and butyric acid as a carbon source for heterotrophic cultivation of oleaginous microalgae such as *C. protothecoides* (Fei et al., 2015). An elevated concentration of acetate in a VFA mixture is believed to favor biomass and lipid accumulation. Microalgae tend to utilize the preferred carbon source and suppress the consumption of other sources, which exhibit diauxic growth. Venkata Mohan and Prathima Devi (2012) suggested that acetate was utilized ahead of butyrate and propionate when a mixed culture of microalgae (*Scenedesmus and Chlorella*) was cultivated in dark fermentation effluents, leading to 1.42 g/L of biomass and 26.4% of lipids under mixotrophic conditions (Venkata Mohan and Prathima Devi, 2012). Liu et al. (2013b) found that, owing to the interaction between organic and inorganic carbon uptake, butyrate removal was higher under heterotrophic than mixotrophic conditions (Liu et al., 2013b). Overall, heterotrophic growth of microalgae on a mixture of organic substrates remains difficult to estimate mainly because the response seems to vary between species and experimental conditions.

**TABLE 2:** Comparative study on various oleaginous microorganisms for their biomass and lipid accumulation after cultivation of mixture of VFAs.

**Microalgal strains**	**Source of VFAs**	**Cultivation conditions**	**Cell dry weight**	**Lipid content**	**References**
*Chlorella vulgaris* UTEX 2714	Acid-rich hydrolysate containing 8.1 g/L of VFAs	3-fold diluted sugar beet pulp with digested dairy manure	2.17 g/L	NA	Wang et al., 2019
Arctic *Chlorella* sp. ArM0029B	Residual NH_4_^+^-N from pretreated coke effluent, supplemented with volatile fatty acids (VFAs)	Stepwise mixotrophic conditions	170.0 ± 7.1 (mg/L/d)	NA	Ryu et al., 2015
*Chlorella sorokiniana* (NCIM No. 5561)	Anaerobically digested wastewater (ADW) of food processing industry	Glucose + ADW	710 ± 14 mg/L	28.8 ± 1.8%	Gupta and Pawar, 2018
		RW + ADW	660 ± 13 mg/L	29.0 ± 2.1%	
*Chlorella zofingiensis*	Mixed biogas slurry (PS) and municipal wastewater (MW)	8%PS + MW indoor	2.5 g/L	21.6%	Zhou et al., 2018
*Chlorella* sp. and *Scenedesmus* sp.	Anaerobic digested piggery effluent (ADPE)	Raceway pond	2.20 ± 0.49gm^–2^d^–1^	6.83%	Moheimani et al., 2018
*Scenedesmus* sp.	Anaerobic digester effluent from palm oil mill	Immobilized microalgae	2.98 g/L	35.92%	Srinuanpan et al., 2019
*Chlorella* sp. L3	Anaerobic sludge (AS)	AS with heat treatment	1.57 g/L		Qi et al., 2017
*Auxenochlorella protothecoides* SAG 211-13	VFAs produced from food waste in an anaerobic digestion membrane bioreactor acetic acid (C2; 2.75 g/L), propionic acid (C3; 1.43 g/L), butyric acid (C4; 1.41 g/L), valeric acid (C5; 0.25 g/L), and caproic acid (C6; 4.78 g/L)	Fresh water heterotrophic cultivation with VFAs and yeast extract (C/N 60)	1.90 g/L	28.97%	This study
*Chlorella sorokiniana* SAG 211-8k (CS)			0.80 g/L	33.79%	
*Aurantiochytrium* sp. T66		Marine water heterotrophic cultivation with VFAs and yeast extract (C/N 10)	1.18 g/L	10.93%	
*Schizochytrium limacinum* SR21			2.23 g/L	5.59%	
*Crypthecodinium cohnii* PGM-1			4.03 g/L	4.26%	

*NA, not available.*

### Effect of VFAs on the Fatty Acid Profile of Freshwater Microalgae Cultured on VFAs at C/N Ratios of 20 and 60

**FIGURE 5:**
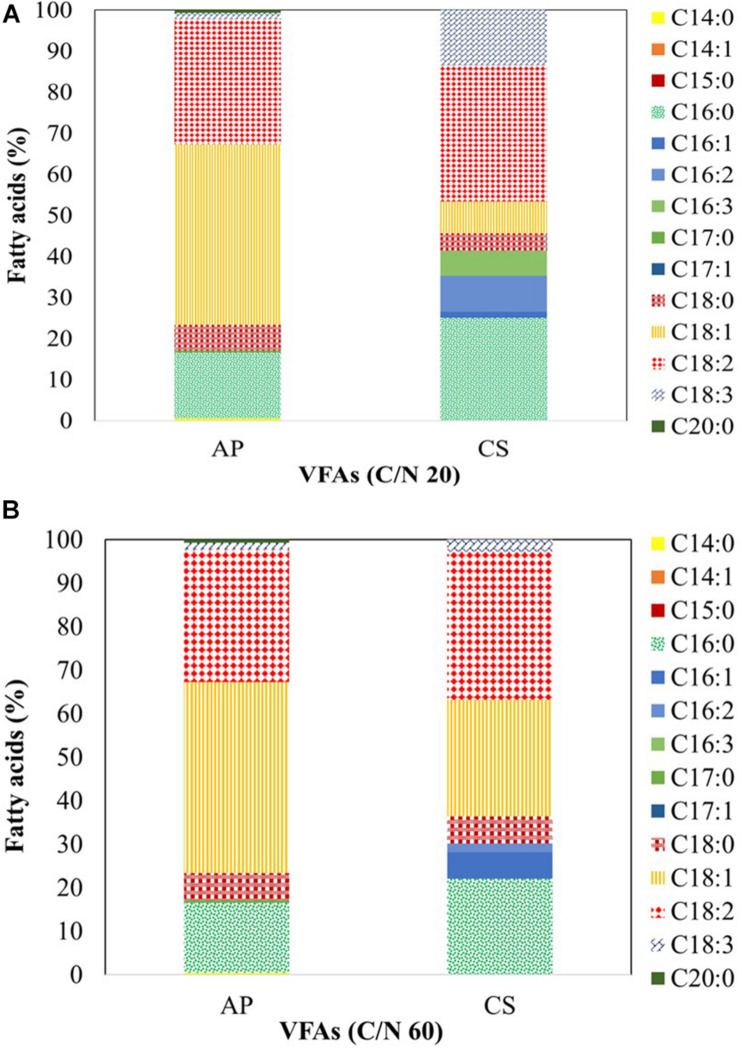
Fatty acid profile of *A. protothecoides* SAG 211-13 (AP) *and C. sorokiniana* SAG 211-8k (CS) cultivated on a VFAs mixture at **(A)** C/N 20 and **(B)** C/N 60. Analysis was carried out by GC-MS. The fatty acids presented as mean value of three independent experiments.

When AP was cultivated on VFAs at C/N of 20 (Figure 5A), it synthesized C14:0 (0.57%), C16:0 (16.05%), C17:0 (0.50%), C18:0 (6.22%), C18:1 (44.01%), C18:2 (30.44%), C18:3 (1.54%), and C20:0 (0.68%). The fatty acid profile changed when the culture was shifted to C/N of 60 (Figure 5B) and included C14:0 (1.90%), C16:0 (23.53%), C17:0 (1.37%), C18:0 (12.24%), C18:1 (45.09%), C18:2 (13.66%), and C18:3 (2.30%). CK cultivated on VFAs at C/N of 20 and 60 showed a distinct profile compared to that of AP which may be due to selective uptake of VFAs. At C/N of 20 (Figure 5A), it synthesized C16:0 (25.10%), C16:1 (1.44%), C16:2 (8.74%), C16:3 (6.04%), C18:0 (4.36%), C18:1 (7.71%), C18:2 (33.08%), and C18:3 (13.53%). At C/N of 60 (Figure 5B), the profile was more similar to that reported with AP at the same C/N, i.e., C16:0 (22.10%), C16:1 (6.04%), C16:2 (2.10%), C18:0 (6.16%), C18:1 (26.87%), C18:2 (34.00%), and C18:3 (2.65%). When VFAs are used for lipogenic fermentation, microbes usually synthesize C16:0, C18:1, and C18:2; these fatty acids are abundant also in soybean and jatropha oil, which makes the product suitable as biodiesel feedstock (Seo et al., 2014). Fatty acid composition plays a key role in the performance of biodiesel (Patel et al., 2020a); comparatively low levels of saturated and polyunsaturated fatty acids but high levels of monounsaturated fats ensure the best compromise between cold flow properties, i.e., the solidification of fuel at lower temperatures, and oxidative stability, i.e., the susceptibility of unsaturated fatty acids to self-oxidation (Patel et al., 2017a). In this respect, AP biomass cultivated on VFAs at C/N of 20 and 60 contained a higher percentage of unsaturated fatty acids than CS.

### Batch Cultivation of Marine Microorganisms on VFAs at C/N Ratios of 10 and 20

**FIGURE 6:**
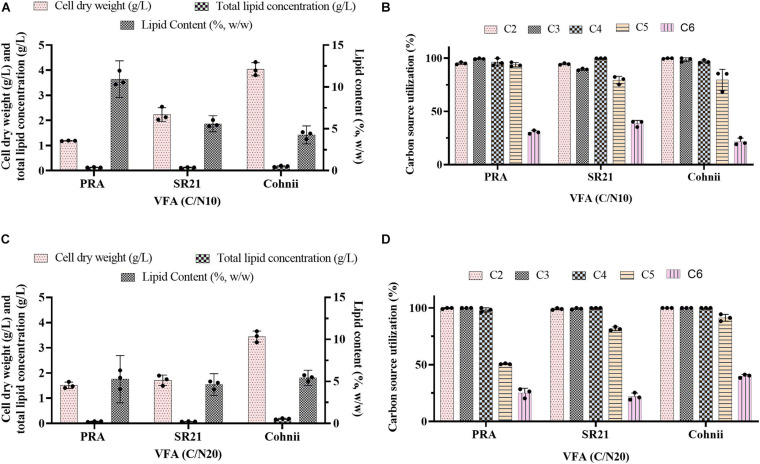
Batch cultivation of the marine thraustochytrids *Aurantiochytrium* sp. T66 ATCC-PRA-276 (PRA) and *S. limacinum* SR21 ATCC-MYA-1381 (SR21), as well as the marine microalga *C. cohnii* PGM-1 ATCC-30772 (Cohnii) on a VFAs solution at **(A,B)** C/N 10 and **(C,D)** C/N 20. **(A,C)** Estimation of cell dry weight (g/L), total lipid concentration (g/L), and lipid content (%, w/w). **(B,D)** Estimation of carbon source utilization. Values represent the average ± standard deviation.

**FIGURE 7:**
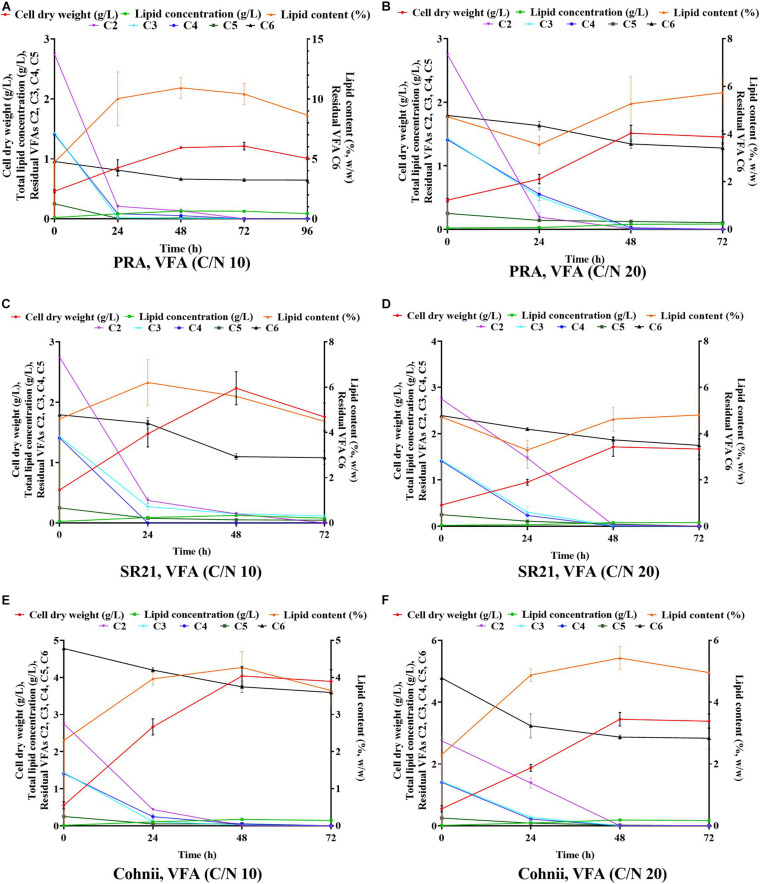
Time course experiment for cell dry weight, lipid concentration, lipid content and residual VFAs when *Aurantiochytrium* sp. T66 ATCC-PRA-276 (PRA) cultivated on VFAs at C/N 10 **(A)** and C/N 20 **(B)**; *S. limacinum* SR21 ATCC-MYA-1381 (SR21), cultivated on VFAS at C/N 10 **(C)** and C/N 20 **(D)**; and *C. cohnii* PGM-1 ATCC-30772 (Cohnii) on a VFAs solution at C/N 10 **(E)** and C/N 20 **(F)**.

In our previous studies, two thraustochytrids strains such as *Schizochytrium limacinum* SR21 and *Aurantiochytrium sp.* T66 (ATCC PRA-276) were successfully cultivated on glucose obtained from forest lignocellulosic biomass for omega-3 production (Patel et al., 2019b, 2020b). However, thraustochytrids were not cultivated on mixture of VFAs previously. Hence, these two thraustochytrids PRA and SR21, as well as the oleaginous marine microalga Cohnii, were cultivated on VFAs at C/N of 10 and C/N 20 (Figures 6A–D). The results of cell dry weight (g/L), total lipid concentration (g/L), and lipid content (%, w/w) are presented in Figures 6A,C; whereas the corresponding carbon source consumption (%) is presented in Figures 6B,D. The time course experiment of cell dry weight, lipid concentration, lipid content and residual VFAs during the cultivation of PRA, SR21 and Cohnii under C/N 10 and C/N 20 are presented in Figure 7. At C/N of 10, PRA cell dry weight, total lipid concentration, and lipid content were 1.19, 0.13 g/L, and 10.93%, respectively (Figure 7A). Almost 95.15% of C2, 99.43% of C3, 96.43% of C4, and 93.88% of C5 were utilized by the time the cell growth reached stationary phase (Figure 7A). The stationary phase was observed at 48 h of cultivation after that decline of biomass was observed till 96 h (Figure 7A). SR21 cell dry weight, lipid concentration, and lipid content were 2.23, 0.12 g/L, and 5.59%, respectively, after utilization of 94.5% of C2, 89.42% of C3, and 99.96% of C4 (Figure 7C). Time course experiment suggested that this strain achieved stationary phase at 48 h of cultivation after utilization of VFAs (C2, C3, C4, C5, and C6). Whereas PRA hardly used C6, SR21 consumed both C5 (79.33%) and C6 (38.66%) from the VFAs solution (Figures 7A,C). The highest cell dry weight (4.03 g/L) and total lipid concentration (0.19 g/L) was achieved by the oleaginous microalgae Cohnii after utilization of 99% of C2, 98.63% of C3, 96.82% of C4, 80% of C5, and 21.63% of C6 (Figures 6A,B).

**FIGURE 8:**
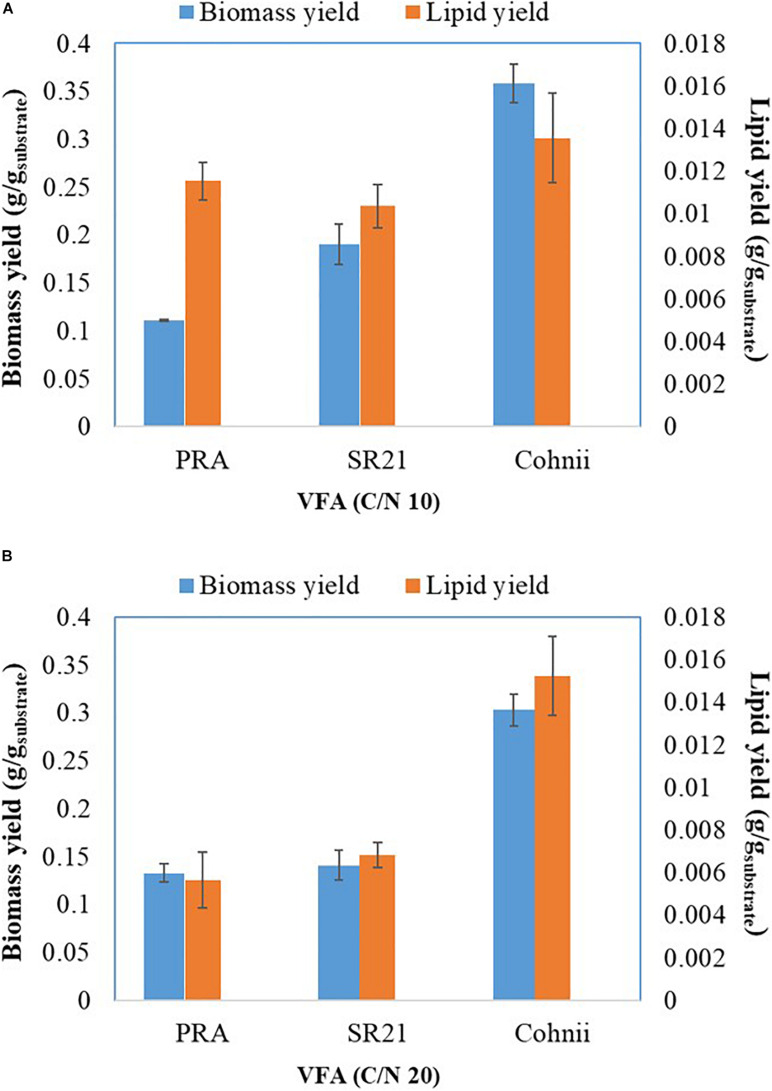
Biomass and lipid yield *of Aurantiochytrium* sp. T66 ATCC-PRA-276 (PRA) and *S. limacinum* SR21 ATCC-MYA-1381 and *C. cohnii* PGM-1 ATCC-30772 (Cohnii) cultivated on VFAs at C/N 10 **(A)** and C/N 20 **(B)**.

After cultivation on VFAs at C/N of 10, the cultivation of marine microorganisms were shifted to C/N of 20 and the effect on growth and lipid accumulation was assessed (Figures 6C,D, 7B,D,F). The cell dry weight of PRA, SR21, and Cohnii was now 1.51, 1.71, and 3.45 g/L, respectively, which is less than what was observed with C/N of 10. The corresponding lipid concentrations were 0.09, 0.078, and 0.18 g/L. In the case of PRA and SR21, these values were lower than those reported with C/N of 10, while the lipid content of Cohnii was nearly identical (Figures 6A,C). These observations may be explained by a lower amount of C5 and C6 being consumed by PRA and SR21 (Figures 7B,D,F). Stationary phase achieved (48 h) by all these microorganisms were similar to those reported with C/N 10 condition, after that no further utilization of VFAs were observed (Figures 7B,D,F). Biomass and lipid yield (g/g_substrate_) for the cultivation of PRA, SR 21 and Cohnii on VFAs at C/N 10 and 20 are presented in Figure 8. PRA cultivated on VFA at C/N 10 showed 0.11 g/g_substrate_ of biomass yield and 0.011 g/g_substrate_ of lipid yield whereas respective values changed to 0.13 g/g_substrate_ of biomass yield and 0.005 g/g_substrate_ of lipid yield when cultivation was shifted from C/N 10 to C/N 60 (Figure 8). In the case of SR 21, the biomass and lipid yield were 0.19 and 0.010 g/g_substrate,_ respectively at C/N 10 where the corresponding values at C/N 20 were 0.14 and 0.006 g/g_substrate_ of lipid yield (Figure 8). The highest biomass (0.36 g/g_substrate_) and lipid yield (0.014) were reported with Cohnii, when cultivated on VFAs at C/N 10, whereas the corresponding value at C/N 20 were 0.30 and 0.015 g/g_substrate_ (Figure 8).

**FIGURE 9:**
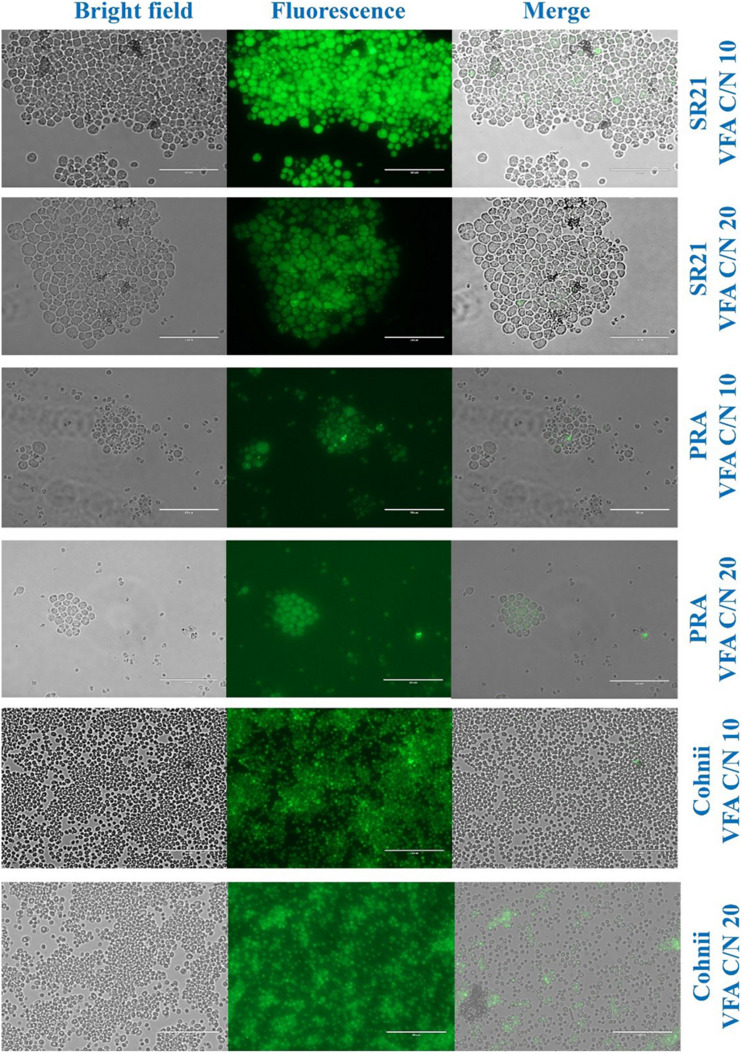
Morphologic analysis of cells and lipid droplets of *Aurantiochytrium* sp. T66 ATCC-PRA-276 (PRA), *S. limacinum* SR21 ATCC-MYA-1381 and *C. cohnii* PGM-1 ATCC-30772 (Cohnii) cultivated on VFAs at C/N 10 and C/N 20. The cells were stained with 4,4-difluoro-1,3,5,7,8-pentamethyl-4-bora-3a,4a-diaza-s-indacene (BODIPY493/503) and observed by live fluorescence microscopy. Scale bars corresponds to 50 μm.

Morphological analysis of cells and lipid accumulation were assessed through fluorescence microscope and the respective images are presented in Figure 9. In the case of SR21, the cell were confined with lipid droplets but in case of PRA, only few cells showed tiny lipid droplets. In Cohnii at C/N10, the lipid droplets were smaller than those reported with C/N 20 (Figure 9).

Few studies on the cultivation of thraustochytrids using single VFA as carbon source have been attempted so far; only *C. cohnii* has been proposed for the production of lipids (Chalima et al., 2017). The oleaginous thraustochytrid *Aurantiochytrium* sp. T66 was cultivated on single VFAs (C1, C2, C3, C4, C5, and C6), which showed that this strain could not utilize C3, C5, and C6 as a substrate if provided at >2 g/L; whereas C2 and C4 could be used efficiently at up to 40 g/L (Patel et al., 2020c). The freshwater microalgae *C. protothecoides* cannot utilize more than 2 g/L of total VFAs (Fei et al., 2015); while some marine microalgae can easily assimilate 30 g/L of acetic acid, 10 g/L of propionate, and 15 g/L of butyric acid (Chalima et al., 2019). *C. cohnii* was cultivated on various VFAs (C2, C3, and C4) at an initial concentration ranging from 5 to 50 g/L; the highest biomass was synthesized on 30 g/L of acetate, 10 g/L of propionate, and 15 g/L of butyrate, while any further increase in the concentration of these substrates caused growth inhibition (Chalima et al., 2019). Therefore, it might be possible that the VFAs mixture provided here to SR21 and Cohnii was converted into lipid-free biomass but was not enough to synthesize any lipids.

### Effect of VFAs on the Fatty Acid Profile of Marine Microorganisms Cultured at C/N Ratios of 10 and 20

**FIGURE 10:**
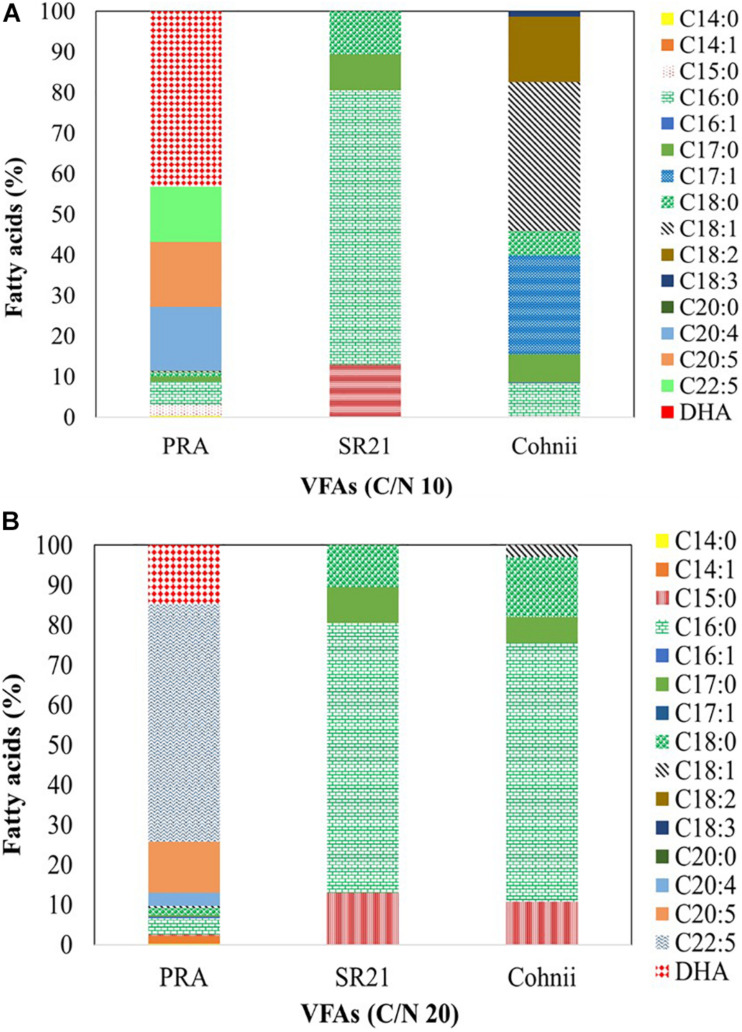
Fatty acid profile of the marine thraustochytrids *Aurantiochytrium* sp. T66 ATCC-PRA-276 (PRA) and *S. limacinum* SR21 ATCC-MYA-1381 (SR21), as well as the marine microalga *C. cohnii* PGM-1 ATCC-30772 (Cohnii) cultivated on a VFAs mixture at **(A)** C/N 10 and **(B)** C/N 20. Analysis was carried out by GC-MS. The fatty acids presented as mean value of three independent experiments.

According to Morabito et al. (2019), C16:0 (palmitic acid) and C22:6 (DHA) constitute more than 65% of total lipids in thraustochytrids. Moreover, the latter display the highest DHA content among various PUFA-producing microorganisms (Morabito et al., 2019). The total amount of lipids and their profile varies between species and genera, with *Aurantiochytrium*, *Thraustochytrium*, and *Schizochytrium* being considered as the most productive genera for DHA (Fossier Marchan et al., 2018). The fatty acid profiles of PRA, SR21, and Cohnii are presented in Figure 10. When PRA was cultivated on VFAs at C/N of 10 (Figure 10A), it generated C14:0 (0.35%), C15:0 (2.77%), C16:0 (5.61%), C17:0 (1.23%), C18:0 (1.08%), C18:1 (0.13%), C20:4 (15.79%), C20:5 (16.03%), docosapentaenoic acid (DPA; 13.56%), and DHA (43.19%). The profile changed dramatically when cells were shifted to C/N of 20 (Figure 10B), whereby fatty acids included C14:0 (0.22%), C14:1 (1.92%), C15:0 (0.46%), C16:0 (3.94%), C16:1 (0.51%), C17:0 (0.43%), C18:0 (1.67%), C20:4 (3.35%), C20:5 (12.81%), DPA (59.42%), and DHA (14.79%). SR21 synthesized mainly C15:0 (12.91%), C16:0 (67.62%), C17:0 (8.92%), and C18:0 (10.55%) when cultivated on a VFAs mixture at C/N of 10 (Figure 10A), and the proportions did not change significantly following a shift to C/N of 20 (Figure 10B), with the corresponding values being C15:0 (10.81%), C16:0 (64.70%), C17:0 (6.57%); C18:0 (14.94%), and C18:1 (2.98%). At C/N of 10 (Figure 10A), Cohnii produced C14:0 (0.17%), C15:0 (0.20%), C16:0 (7.94%), C17:0 (6.94%), C17:1 (24.30%), C18:0 (6.08%), C18:1 (36.70%), C18:2 (16.06%), and C18:3 (1.35%). The profile did not change significantly when Cohnii was shifted to C/N of 20 (Figure 10B). In our previous study, cultivation of PRA on individual VFAs (formic acid, acetic acid, propionic acid, butyric acid, valeric acid, and caproic acid) at 2 g/L, impeded the synthesis of DPA and DHA (Patel et al., 2020c). However, when the strain was cultivated on >2 g/L VFAs, it produced omega-3 fatty acids in all cases except with C4 and C6 at 10 g/L (Patel et al., 2020c). Overall, PRA is the most promising among the tested marine microorganisms for the synthesis of high amounts of DPA and DHA at both C/N ratios.

Although both SR21 and Cohnii are known PUFA producers, they were unable to synthesize fatty acids longer than C18:3, however, the mechanisms is still unknown with VFAs as substrates. In the photosynthetic microorganisms such as microalgae, the *de novo* fatty acid synthesis occurs in plastids whereas in non-photosynthetic microorganisms such as thraustochytrids, it occurs in cytoplasm. PUFA synthesis enzymes such as desaturases and elongases are confined at endoplasmic reticulum (ER) membrane (Tehlivets et al., 2007). The final product of fatty acid synthase (FAS) is always C18:0 (stearic acid) that can be converted into C18:1 (oleic acid) by stearoyl-acyl carrier protein (ACP) Δ9-desaturase or acyl-CoA Δ9-desaturase depending on substrate availability. All PUFA producing microorganism including higher plants have Δ 12-, and Δ 15-desaturases to convert oleic acid into linoleic acid (LA, 18:2^Δ9,12^) and α-linolenic acid (ALA, 18:3^Δ9,12,15^), while animals and human lack these enzymes (Gong et al., 2014). The conversion of LA and ALA into long chain PUFA need involvement of multiple front-end desaturases, C18- and C20-PUFA-specific elongases (Pereira et al., 2004). PUFAs can be synthesized in these microorganisms through two different pathways: an oxygen-independent pathway known as PUFA synthase pathway or anaerobic polyketide synthase pathway, and an oxygen-dependent pathway or aerobic fatty acid synthase pathway also known as elongase-desaturase pathway (Qiu, 2003). In the latter, PUFA synthesis is initiated with C18:3^Δ9,12,15^, which is first converted to C18:4^Δ6,9,12,15^ by Δ6 desaturase, then to C20:4^Δ8,11,14,17^ by elongase, and to C20:5^Δ5,8,11,14,17^ by Δ5 desaturase. Finally, elongase is responsible for conversion to 22:5^Δ7,10,13,16,19^ and Δ4 desaturase for the generation of DHA (22:6^Δ4,710,13,16,19^) (Qiu, 2003; Sijtsma and De Swaaf, 2004; Monroig et al., 2012; Morabito et al., 2019). Hence, PUFA synthesis can only proceed with sufficient C18:3 as a substrate. An alternative to desaturase/elongase system for PUFA production, known as anaerobic polyketide synthase (PKS) is present in both eukaryotes and prokaryotes. Isolation of Δ4-desaturase from the eukaryotic marine thraustochytrid *Thraustochytrium* sp., indicated the role of aerobic desaturase–elongase system for PUFA synthesis. However, Metz et al. (2001) suggested after cDNA sequencing of *Schizochytrium* that the 8,500 ESTs region do not include with expected number of desaturases, whereas this sequence are more a like to *Shewanella* PKS-like ORFs. The cloning of *Schizochytrium* PKS-like genes with three ORFs showed similarities with five ORFs from *Shewanella*, which indicates a strong evolutionary association between the two species by the shared existence of conserved PKS domains. It is also possible that a PKS-like mechanism occurs in some lower eukaryotes as well as in bacteria for the biosynthesis of PUFAs (Gong et al., 2014). Both the enzymatic system e.g., aerobic desaturase–elongase system and PKS system co-exists in *Schizochytrium* (Metz et al., 2001) and *Thraustochytrium* (Qiu et al., 2001). While both the traditional aerobic pathway and the anaerobic PKS-like pathway in both microorganisms are present, *Schizochytrium* lacks the action of Δ12-desaturase that cannot synthesize PUFA from the conventional FAS pathway. This observation indicate that *Schizochytrium* sp. has partial framework of desaturase/elongase (Lippmeier et al., 2009), whereas *Thraustochytrium aureum* have functional Δ12-desaturase that can produce ω3 very long chain PUFAs via the conventional desaturase/elongase pathway and the PKS-like pathway (Gong et al., 2014). Such findings indicate that microbes include either the anaerobic PKS-like pathway or the traditional aerobic desaturase/elongase pathway, or both for PUFA biosynthesis. In a study De Swaaf et al. (2003a) concluded after 13C labeling research that *C. Cohnii* doesn’t involve the role of desaturase for DHA biosynthesis (De Swaaf et al., 2003a).

## Conclusion

Two fresh water oleaginous microalgae for biofuel production and one marine microalga including two marine thraustochytrids for omega-3 production were explored to assimilate the VFAs produced from food waste via anaerobic digestion in membrane bioreactor. Both fresh water microalgae *A. protothecoides* SAG 211-13 (AP) *and C. sorokiniana* SAG 211-8k (CS) were synthesized 28.97 and 33.79% of lipids in their cellular compartments that can be used as biodiesel feed stock due to similar fatty acids profile to vegetable oils. Among the marine microorganisms, marine thraustochytrids *Aurantiochytrium* sp. T66 ATCC-PRA-276 synthesized 1.19 g/L of lipids in which amounts of DPA and DHA were 13.56 and 43.19%, respectively. Other marine microalga *Crypthecodinium cohnii* PGM-1 and thraustochytrids *S. limacinum* SR21 were unable to produce high amount of lipids with higher chains omega-3 fatty acids due to lacking of proper amount of substrates in the form of VFAs mixture. From this study, we can conclude that not only the amount of VFAs but also their ratio in the mixture affect the assimilation for the growth and lipid accumulation by various type of oleaginous microorganisms. Further study is required to figure out the effect of the amount of individual VFAs and their different ratio in a mixture on the growth of the selected oleaginous microorganisms.

## Data Availability Statement

The original contributions presented in the study are included in the article/supplementary material, further inquiries can be directed to the corresponding author/s.

## Author Contributions

AP designed and performed the experiments, analyzed the data, and drafted the manuscript. AM produced the effluents, contributed to data analysis, and revised the manuscript. IH and MT contributed to the idea development, provided the effluents, and revised the manuscript. UR and PC conceived the study, discussed the results, and revised the manuscript. LM conceived the study, contributed to the experimental design and data analysis, discussed the results, and contributed in drafting the manuscript. All the authors discussed the results and commented on the manuscript.

## Conflict of Interest

The authors declare that the research was conducted in the absence of any commercial or financial relationships that could be construed as a potential conflict of interest.
